# Nighttime sleep benefits the prospective component of prospective memory

**DOI:** 10.3758/s13421-021-01187-w

**Published:** 2021-06-11

**Authors:** Mateja F. Böhm, Ute J. Bayen, Reinhard Pietrowsky

**Affiliations:** Institute for Experimental Psychology, Heinrich-Heine-Universität Düsseldorf, Universitätsstraße 1, Geb. 23.02., 40225 Düsseldorf, Germany

**Keywords:** Prospective memory, Intention, Sleep, Multinomial modeling

## Abstract

Studies suggest that sleep benefits event-based prospective memory, which involves carrying out intentions when particular events occur. Prospective memory has a prospective component (remembering *that* one has an intention), and a retrospective component (remembering *when* to carry it out). As effects of sleep on retrospective memory are well established, the effect of sleep on prospective memory may thus be due exclusively to an effect of sleep on its retrospective component. Therefore, the authors investigated whether nighttime sleep improves the prospective component of prospective memory, or a retrospective component, or both. In a first session, participants performed an event-based prospective-memory task (that was embedded in an ongoing task) 3 minutes after forming an intention and, in a second session, 12 hours after forming an intention. The sessions were separated by either nighttime sleep or daytime wakefulness. The authors disentangled prospective-memory performance into its retrospective and prospective components via multinomial processing tree modeling. There was no effect of sleep on the retrospective component, which may have been due to a time-of-day effect. The prospective component, which is the component unique to prospective memory, declined less strongly after a retention interval filled with sleep as compared with a retention interval filled with wakefulness. A hybrid interaction suggested that refreshed attention after sleep may account for this effect, but did not support the consolidation of the association between the intention and its appropriate context as a mechanism driving the effect.

**Supplementary Information:**

The online version contains supplementary material available at 10.3758/s13421-021-01187-w.

## Introduction

The beneficial effect of sleep on memory for past events (*retrospective memory*) has been thoroughly investigated and firmly established (for a review, see Rasch & Born, 2013). Recent studies suggest that sleep also benefits *prospective memory* (PM; for a review, see Leong, Cheng, et al., 2019). Event-based PM involves carrying out intentions when particular events occur and is an important and ubiquitous type of task in daily life (for an overview of the field of PM research, see Bayen et al., 2019). For instance, diabetics may have to remember to take insulin when being served a meal. PM has two components (Einstein & McDaniel, 1990). The prospective component involves remembering *that* an intention must be carried out, whereas the retrospective component involves remembering *what* must be done and *when*.

### Effects of sleep on PM

Total sleep deprivation decreases PM performance (Grundgeiger et al., 2014), whereas sleep that takes place during the retention interval improves PM performance (Barner et al., 2017; Diekelmann et al., 2013a, 2013b; Leong, Koh, et al., 2019; Leong, van Rijn, et al., 2019; Scullin & McDaniel, 2010). Some studies suggested that objective sleep quality, total sleep time, and specific sleep stages play an important role in this positive effect of sleep on PM (Diekelmann et al., 2013b; Fabbri et al., 2014; Kyle et al., 2017; Leong, Koh, et al., 2019; Scullin et al., 2019). While previous research has mostly been dedicated to the specific properties of sleep that may improve PM, it remains unclear which components of PM benefit from sleep. Our study set out to answer this question while also trying to disentangle possible mechanisms that may contribute to effects of sleep on prospective and retrospective components of PM.

#### Mechanisms of sleep effects

There are three different theoretical mechanisms by which sleep may differentially benefit the prospective and the retrospective components of PM.

##### First mechanism of sleep effects: Retrospective-memory consolidation

*Memory consolidation* describes the transition of information in memory from an unstable to a stable state, meaning that information is more resistant to forgetting after consolidation (e.g., Stickgold & Walker, 2005). Sleep is a “brain state optimizing memory consolidation” (Rasch & Born, 2013), leading to a retrospective-memory benefit after sleep as compared with wakefulness. Importantly, in order for retrospective-memory content to benefit from consolidation, sleep must take place during the retention interval between encoding and retrieval of said content.

##### Second mechanism of sleep effects: Consolidation of the intention–context association

Scullin and McDaniel (2010) proposed that in PM tasks, sleep consolidates the link between the intention and its appropriate context (*intention–context association*). For example, if you intend to buy milk at the store, sleep between the formation of the intention and the opportunity for its execution may strengthen the association between your intention (buying milk) and its context (the store) so that you are more likely to retrieve the intention in the appropriate context. This leads, in turn, to the strategic involvement of attentional processes to monitor for PM targets in this context (Scullin et al., 2013). As the initiation of monitoring is dependent on the retrieval of the intention–context association, participants can initiate monitoring only if they remember their intention when they find themselves in the relevant context. Hence, stronger intention–context associations following sleep should enhance the prospective component of PM. Critically, in order for the prospective component to benefit from a consolidated intention–context association, sleep has to take place *during* the retention interval.

##### Third mechanism of sleep effects: Refreshed attention

Prolonged wakefulness impairs sustained attention (Schmidt et al., 2007). After sleep, attention is refreshed. If the prospective component of PM tasks relies on attentional processes, it should benefit from sleep that occurs shortly before the PM task as compared with longer periods of wakefulness. Critically, in order for PM to benefit from refreshed attention, sleep does *not* have to take place during the retention interval, but may also take place before the encoding of the PM intention.

## The design and task of the current experiment

A laboratory event-based PM task is typically embedded in an ongoing computerized two-answer forced-choice task (Einstein & McDaniel, 1990). After receiving instructions for the ongoing task and practicing it, participants receive PM-task instructions. For the PM task, they are instructed to remember to press a particular key whenever certain events occur during the ongoing task. Such events may be the occurrence of PM target words during the ongoing task. The PM instructions are typically followed by a retention interval, in which participants perform unrelated tasks, to prevent ceiling effects in the PM task. Participants then perform the ongoing task with the embedded PM task.

**Fig. 1:**
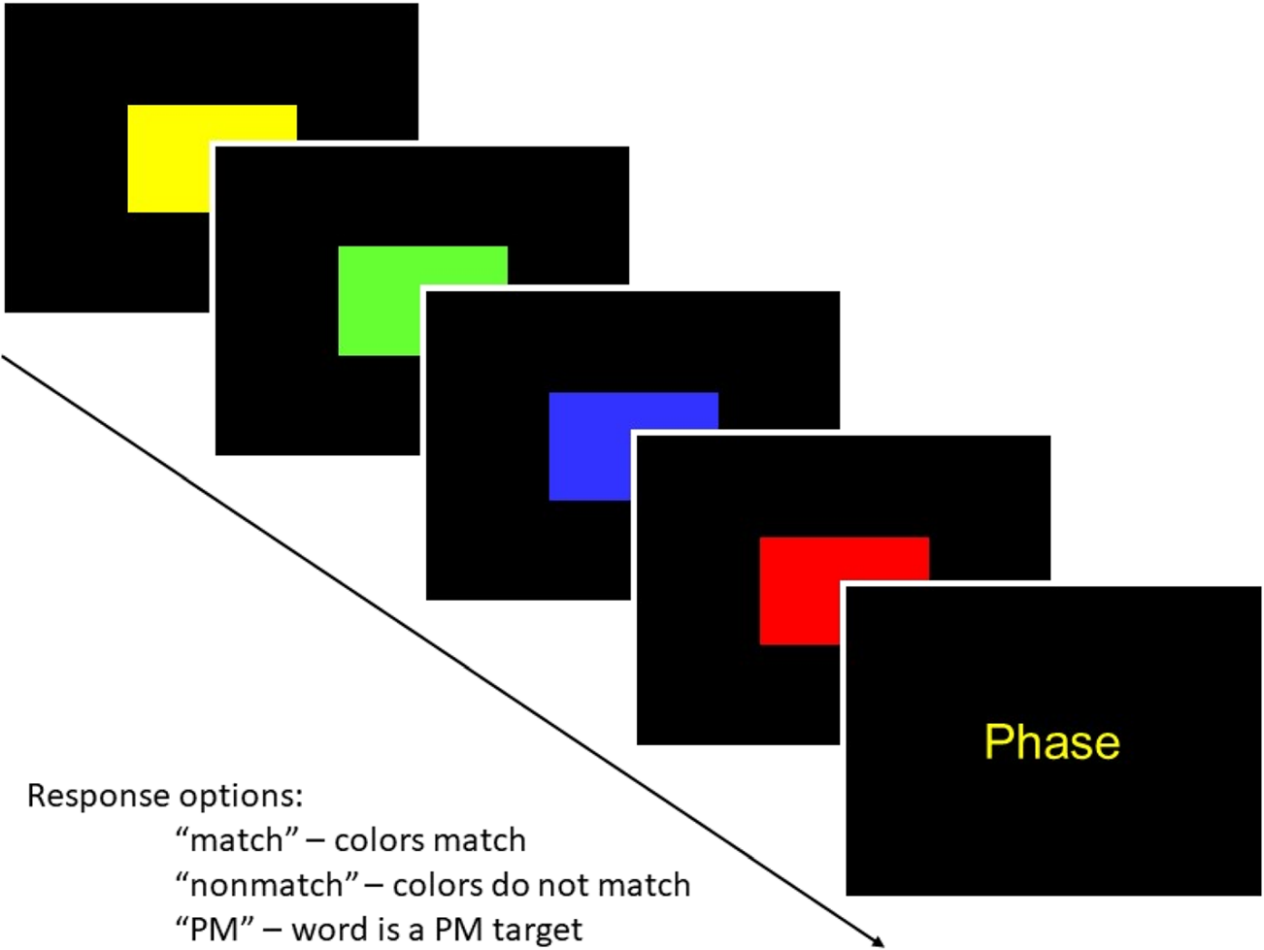
An example trial of the ongoing color-matching task with the embedded prospective-memory task. *Note.* PM = prospective memory. Adapted with permission from “Prospective Memory: Adult Age, Ongoing Task Difficulty, and Task Importance [Poster presentation],” by Smith & Hunt, 2012, April, Biannual Cognitive Aging Conference, Atlanta, GA, USA

Figure 1 illustrates the task we used in our computer-based experiment. As shown, the ongoing task was to indicate color match or nonmatch between a series of rectangles and a subsequently presented word via key press. The embedded PM task was to press a different key whenever one of several previously studied PM target words appeared (cf. Smith & Bayen, 2004). As the third possible mechanism of sleep effects on PM was refreshed attention, we chose a PM task that required attentional resources—namely a *nonfocal* PM task. The shown PM task is nonfocal to the ongoing task because the task requires shifting the attentional focus from the characteristics of the ongoing task (word color) to the PM task (word meaning). In nonfocal PM tasks, the prospective component thus requires attentional processes (e.g., McDaniel & Einstein, 2000; Scullin et al., 2013; Smith, 2003; for a meta-analysis, see Anderson et al., 2019). The retrospective component of *when* to press the PM key requires recognizing PM targets (i.e., discriminating them from distractor words).

**Fig. 2:**
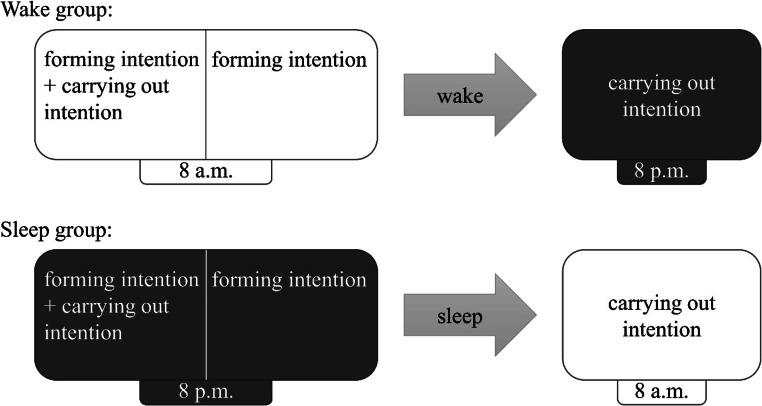
Study design

Figure 2 illustrates the experimental design. Participants were assigned to a wake group or a sleep group. All participants completed one session at 8 a.m. and one at 8 p.m. In the wake group, the first session was in the morning and the second session in the evening, and vice versa in the sleep group. In the first session, participants were instructed to perform a PM task during the ongoing task, which started after a 3-minute retention interval. Immediately after completion of the task (i.e., also in the first session), participants received PM instructions for the second session and new target words for the second session. The corresponding task was completed in the second session, which took place after a 12-hour retention interval. In the wake group, participants were awake during the 12-hour retention interval. For the sleep group, session timing was reversed such that participants slept during the 12-hour retention interval.

## Measuring the prospective and the retrospective component of PM

Sleep may benefit either or both components of PM via three different theoretical mechanisms as outlined above. To disentangle these different mechanisms, it is essential to obtain separate and unconfounded measures of the prospective and the retrospective component of PM. To this end, we used a stochastic modeling approach: The multinomial processing tree (MPT) model of event-based PM (Smith & Bayen, 2004) allows us to obtain separate, statistically independent measures of the prospective and retrospective component of PM in the type of task illustrated in Fig. 1. A detailed description of the MPT model is in the Analyses and Results section. The model assumes that with probability *P* (prospective component), participants remember that they must do something in addition to the ongoing task. If they remember this, then with conditional probability *M* (retrospective component), they will recognize a PM target event when it occurs. Parameters *P* and *M* are thus independent measures of the prospective and retrospective components of PM, respectively, and can therefore be used to disentangle effects of sleep on both components.

## Hypotheses

Different hypotheses can be derived from the three described theoretical mechanisms that may underlie sleep effects on PM. The hypotheses are explained in turn and listed in Table 1.

**Table 1:** The mechanisms of sleep effects on PM and the hypotheses derived

Mechanism	Dependent variable		Hypothesis
Retrospective-memory consolidation	Retrospective component of PM (model parameter *M*)	1a	No group difference in Session 1.
		1b	Sleep group better than wake group in Session 2.
		1c	Decline from Session 1 to Session 2 in both groups.
		1d	Weaker decline from Session 1 to Session 2 in the sleep than in the wake group.
		1e	Ordinal interaction.
Consolidation of the intention–context association	Prospective component of PM (model parameter *P*)	2a	No group difference in Session 1.
		2b	Sleep group better than wake group in Session 2.
		2c	Decline from Session 1 to Session 2 in both groups.
		2d	Weaker decline from Session 1 to Session 2 in the sleep than in the wake group.
		2e	Ordinal interaction.
	Number of participants never pressing the PM key	3a	No group difference in Session 1.
		3b	Higher number in the wake group than the sleep group in Session 2.
		3c	Increase from Session 1 to Session 2 in both groups.
		3d	Weaker increase from Session 1 to Session 2 in the sleep than in the wake group.
Refreshed attention	Prospective component of PM (model parameter *P*)	4a	Wake group better than sleep group in Session 1.
		4b	Sleep group better than wake group in Session 2.
		4c	Decline from Session 1 to Session 2 in both groups.
		4d	Weaker decline from Session 1 to Session 2 in the sleep than in the wake group.
		4e	Hybrid interaction.

*PM* prospective memory.

### Hypotheses Series 1 derived from the retrospective-memory-consolidation account

As a result of retrospective-memory consolidation, sleep during the retention interval should strengthen the memory representation of PM targets such that participants recognize PM targets more easily after sleep than after wakefulness. Thus, sleep should benefit the retrospective recognition component of PM after the retention interval. In our experiment, we expected no difference between participant groups in the first session (Hypothesis 1a), because the retention interval in this session was only 3 minutes during which neither group slept. In the second session, however, the sleep group should benefit from the 12-hour retention interval during which sleep took place. In this second session, the sleep group should, therefore, show a better retrospective component than the wake group (Hypothesis 1b).

Furthermore, the retrospective component of PM should decline from the first to the second session due to the differing lengths of the retention intervals (Hypothesis 1c). Yet, in the sleep group, PM-target consolidation should counteract this decline. Thus, we predicted an *ordinal* interaction (Hypothesis 1e) with similar levels of the retrospective component in the first session, followed by a weaker decline in the sleep than the wake group (Hypothesis 1d).

Given the well-established effect of sleep on retrospective memory, it is possible that the sleep effect on overall PM performance is solely due to a positive effect of sleep on the retrospective component of PM. However, sleep may benefit the prospective component of PM by the other two mechanisms described above, implying that the prospective component may also contribute to a sleep benefit to overall PM performance.

### Hypotheses Series 2 and 3 derived from the consolidation of the intention–context association account

If the intention–context association is consolidated, sleep during the retention interval should benefit the prospective component of PM. In this case, we expect no difference in the prospective component between both groups in the first session (Hypothesis 2a) because of the short retention interval filled with wakefulness. In the second session, however, the sleep group should benefit from sleep consolidating the intention–context association, leading to a better prospective component in the sleep group (Hypothesis 2b). As the intention–context association should become less retrievable over time, we expected a decrease in the prospective component of PM after longer retention intervals. Thus, the prospective component should decrease from the first to the second session in both groups (Hypothesis 2c). Sleep during the retention interval, however, may consolidate the intention–context association, thereby facilitating its retrieval in the second session. Sleep should thus counteract the expected decline in the prospective component from the first to the second session. This pattern also constitutes an *ordinal* interaction (Hypothesis 2e): From the first to the second session, the prospective component is expected to decline less in the sleep group than in the wake group (Hypothesis 2d).

In addition, the ability to retrieve the intention–context association should affect the number of participants forgetting to press the PM key altogether. We did not expect a difference between these numbers in the first session (Hypothesis 3a). In the second session, however, we expected more participants in the wake group than the sleep group to forget to press the PM key altogether because the sleep group would benefit from a consolidated intention–context association (Hypothesis 3b). However, the number of participants forgetting to press the PM key altogether should increase from the first to the second session in both groups (Hypothesis 3c), as a result of the intention–context association’s decreased retrievability after the long retention interval. As sleep would counteract this decreased retrievability of the intention–context association, the number of participants who never press the PM key should increase less in the sleep group than in the wake group (Hypothesis 3d).

### Hypotheses Series 4 derived from the refreshed-attention account

Refreshed attention after sleep should benefit the prospective component of PM in the morning, regardless of whether participants slept during the retention interval (sleep group) or before their first session (wake group). Therefore, from a refreshed-attention account, we can derive the hypotheses that in the first session, the wake group should fare better in the prospective component than the sleep group (Hypothesis 4a), whereas in the second session, the sleep group should fare better than the wake group (Hypothesis 4b). At the same time, there would be a main effect of session number on the prospective component, with both groups showing a decline from the first to the second session due to the longer retention interval (Hypothesis 4c, which is the same as 2c above). This overall pattern of results would constitute a *hybrid* interaction with a globally interpretable main effect of session and main effects of group in different directions depending on session (Hypothesis 4e).

Critically, we expected different types of interaction in the prospective component, depending on the mechanisms underlying sleep effects: an ordinal interaction with consolidation of the intention-context association (Hypotheses 2e), and a hybrid interaction with refreshed attention (Hypothesis 4e).

There is a third possibility—namely, that sleep exerts an effect on the prospective component via both refreshed attention *and* consolidation of the intention–context association. This would result in a hybrid interaction as well: In the first session, the wake group would benefit from refreshed attention, resulting in a better prospective component in the wake group than the sleep group. In the second session, the sleep group would benefit both from refreshed attention as well as a consolidated intention–context association, resulting in a better prospective component in the sleep group than the wake group. Critically, we would expect different effect sizes of the group differences in both sessions depending on whether refreshed attention alone benefits the prospective component or whether consolidated intention–context associations also contribute to the effect: Effects of sleep via refreshing of attention alone would lead to equal group differences in the prospective component in both sessions, albeit in different directions (Hypotheses 4a and 4b above). If, however, a combination of both refreshing of attention during sleep and consolidation of the intention–context association during sleep accounted for the interaction, we would expect the superiority of the sleep group over the wake group in the second session to be larger than the superiority of the wake group over the sleep group in the first session. This is because in the morning (i.e., in their second session), the participants in the sleep group would benefit from both refreshed attention as well as sleep-induced consolidation of the intention–context association.

## Method

### Participants

The Chair of the Research Ethics Committee of the College of Mathematics and Natural Science of the Heinrich-Heine-Universität Düsseldorf waived review for this study. Participants were university students who were compensated with course credit or money. Inclusion criteria were screened via online self-report to ensure normal sleep behavior and stimulus processing: German native speaker, 18 to 30 years old, no achromatopsia, no travel to time zones differing more than 3 hours in the last month, no shift work in the last month, no regular sleep medication, no sleep disorders or regular nightly awakenings, no neurological or psychological disorders, no alcohol dependency, no regular recreational drug use, not pregnant, no previous participation in PM studies. We selected participants who followed a regular sleep schedule and would be able to sleep at least 7 hours between sessions if assigned to the sleep group. Participants had to be willing to participate in either experimental group to avoid selective dropout. They refrained from consumption of alcohol for 24 hours and of recreational drugs for 48 hours prior to and for the duration of the study.

We determined the required sample size by performing a power analysis with multiTree (Moshagen, 2010), a computer program for multinomial modeling. To find interactions in PM components with small effect sizes of ω = .024, an alpha of .05, and a power of .80, we needed 31 participants per group with 110 trials per session. Participants were alternatingly assigned to the sleep or wake group, depending on the temporal order in which they participated in the screening for inclusion criteria. One participant in the wake group was replaced because he did not return for the second session. Sample sizes were 31 per group (after exclusion of two participants whose data were lost due to technical error), ranging in age between 18 and 29 years (*Mean* = 22.74, *SD* = 2.77). Six participants in the sleep group and five in the wake group were male, the others female.

### Design

Figure 2 illustrates the 2 × 2 design with between-subjects factor “group” (wake, sleep) and within-subjects factor “session number” (first, second). The wake group completed the first session in the morning, the second in the evening; the sleep group vice versa. Dependent variables were overall PM performance (operationalized as PM hit rate, which is the proportion of PM trials correctly responded to), the model-based parameters for the prospective and retrospective components of PM performance, and the proportion of participants who pressed the PM key at least once. In addition to the experimental tasks, we administered several sleep-related measures before and during the study to ensure equivalence across experimental groups.

### Measurement instruments and materials

#### Sleep diary

We used the Consensus Sleep Diary–Core (Carney et al., 2012), which has been thoroughly validated (Maich et al., 2018). It consists of eight questions and a comment section for each day of the week. The first seven questions are open questions pertaining to the time participants went to sleep (i.e., at what time they went to bed, tried to sleep, and fell asleep), their awakenings (frequency and lengths), and the time they finally awakened and got out of bed. On the eighth item, participants rate their subjective sleep quality on a 5-point scale from *very poor* to *very good*. From these data, we obtained information about total sleep time, subjective sleep quality, and sleep efficiency.

#### Karolinska Sleepiness Scale

The Karolinska Sleepiness Scale (KSS; Åkerstedt & Gillberg, 1990) is a single item, on which participants indicate their current level of sleepiness on a 9-point scale ranging from 1 (*extremely alert*) to 9 (*extremely sleepy–fighting sleep*). We implemented a computerized version of the KSS.

#### Morningness-Eveningness Questionnaire

We assessed chronotype because preferred time-of-day can affect cognitive functioning (Schmidt et al., 2007). The Morningness-Eveningness Questionnaire (MEQ; Horne & Östberg, 1976) consists of 19 items capturing respondents’ preferred time of day. The answers to each item are assigned scores that are summed up to a total score that can range between 14 and 86 and indicates whether participants are morning, evening, or intermediate chronotypes, with higher scores indicating greater morningness.

#### Materials for the prospective-memory and ongoing color-matching tasks

We chose a total of 220 words, which were to be presented after the rectangles during the ongoing color-matching task. To avoid material effects, we created four lists of 55 words each. From each list, we chose five words as PM targets; the other words served as distractors. The lists as well as targets and distractors did not differ in concreteness, arousal, valence, frequency, word length, and syllable count (according to Heister et al., 2011; Lahl et al., 2009). Each word was presented twice, resulting in 110 trials per list. Half of the participants of each group received Lists A and B (counterbalanced across sessions), the other half Lists C and D (also counterbalanced).

### Procedure

For the week prior to participation, participants completed the sleep diary at home every morning.

#### Session 1

Within group, up to four participants were tested simultaneously. They signed informed consent and returned their sleep diary. They then indicated their current sleepiness on the KSS.

Then, participants read the instructions for the computer-based ongoing color-matching task, in which they indicated whether the color of a word matched that of four previously presented rectangles. Possible colors were red, blue, green, yellow, and white. Each rectangle was presented for 500 ms, with interstimulus intervals of 250 ms. The colors were presented equally often. Matches and nonmatches occurred equally often. Participants used the “v” key and the “m” key on their computer keyboard for self-paced “match” and “nonmatch” responses. Assignment of keys to response options was approximately counterbalanced. Six practice trials of the ongoing task were followed by PM instructions. Participants were asked to press the space bar instead of the “match” or “nonmatch” key whenever they encountered one of five PM targets. They were told that they could press the space bar even after responding “match” or “nonmatch.” Then, the five PM targets were presented in random order in black Arial 24 on white background for 5 seconds each.

During the 3-minute retention interval following target presentation, participants solved simple arithmetic equations with feedback. Then, they completed 110 trials of the color-matching task, 10 of these with PM targets (occurring on every 9th to 13th trial, 11th on average). To allow a belated PM response to the last PM target, we added one trial at the end. If participants pressed the space bar on this trial, this counted as a belated PM response. If not, this trial was removed from analyses. After the task, participants were told that they would now study five new words for the same type of embedded PM task to be completed in the second session. The targets from the second list were then presented in the same manner as the previous targets. Participants again solved arithmetic equations for 3 minutes.

The wake group was asked to refrain from daytime napping. The sleep group spent the night at home and completed the sleep diary at home in the morning.

#### Session 2

Participants returned to the laboratory after 12 hours and completed the ongoing task with the PM task at the computer. They were not reminded of the PM task. After completing the task, they indicated whether they knew that they were supposed to do another task in addition to the ongoing task. They further indicated which key they had to press when encountering a PM target.

Participants were then given a paper-based questionnaire to double-check inclusion criteria and ask about target rehearsal during the retention interval. Furthermore, they completed the MEQ. Finally, participants were debriefed and compensated.

## Analyses and results

All data are available via the Open Science Framework (https://osf.io/83bdn/). 

### Tests of equivalence of experimental groups

#### Sleep diary

Table 2 shows descriptive data. We performed multivariate analyses of variance (MANOVAs), with subjective sleep quality, total sleep time, and sleep efficiency (i.e., the ratio of minutes in bed that were spent asleep) as dependent variables. There were no between-group differences in the week prior to participation, Pillai’s trace = .09, *F*(3, 58) = 1.99, *p* = .125, η_p_^2^ = .09. One participant was excluded from analyses involving the sleep group’s experimental night because of missing data. There was a difference between the sleep group’s experimental night and their usual sleep, Pillai’s trace = .55, *F*(3, 27) = 10.80, *p* < .001, η_p_^2^ = .55, which was due to less total sleep time during the experimental night, *F*(1, 29) = 21.72, *p* < .001, η_p_^2^ = .43. However, the sleep group’s experimental night and the wake group’s night before their first session did not differ, Pillai’s trace = .13, *F*(3, 56) = 2.71, *p* = .054, η_p_^2^ = .13, suggesting that the shorter sleep was due to having an early appointment at the university.

**Table 2:** Means and 95% confidence Intervals of the control measures and ongoing-task performance for the sleep group and the wake group

Measure		Sleep group	Wake group
One-week sleep efficiency		83.51 [81.04, 85.98]	85.46 [82.98, 87.94]
One-week sleep quality^a^		2.80 [2.61, 2.98]	2.71 [2.51, 2.92]
One-week average nightly total sleep time in hours		7.68 [7.32, 8.04]	7.50 [7.25, 7.75]
One-night sleep efficiency^b^		84.79 [81.76, 87.81]	87.76 [84.15, 91.38]
One-night sleep quality^a, b^		2.57 [2.31, 2.82]	2.77 [2.42, 3.12]
One-night total sleep time in hours^b^		6.89 [6.56, 7.22]	6.24 [5.50, 6.97]
MEQ		49.74 [47.46, 52.02]	52.52 [49.49, 55.54]
KSS		4.39 [3.80, 4.98]	4.03 [3.32, 4.74]
Number of rehearsals		2.52 [1.99, 3.04]	3.13 [1.31, 4.95]
Rehearsal duration in min.		3.97 [2.48, 5.46]	4.42 [1.09, 7.74]
Ongoing-task percent correct	Session 1	.85 [.82, .88]	.83 [.80, .86]
	Session 2	.88 [.85, .91]	.85 [.82, .89]
Ongoing-task reaction times in ms	Session 1	1,398 [1,268, 1,527]	1,480 [1,338, 1,621]
	Session 2	1,256 [1,165, 1,347]	1,240 [1,069, 1,410]

*MEQ* Morningness-Eveningness Questionnaire, *KSS* Karolinska Sleepiness Scale. ^a^ Higher values indicate better sleep quality. ^b^ Night before the second session (sleep group) or night before the first session (wake group). 95% confidence intervals are in brackets

#### Morningness-Eveningness Questionnaire

Groups did not differ in MEQ total scores, *t*(60) = −1.50, *p* = .140, *d* = .38, which identified them as intermediate chronotypes on average.

#### Karolinska Sleepiness Scale

Groups did not differ in first-session KSS ratings, *t*(60) = 0.78, *p* = .436, *d* = .20.

#### Rehearsal

Groups did not differ in proportion of participants rehearsing targets, *z* = 1.82, *p* = .069, *d* = .48, with 27 rehearsing in the sleep group and 21 in the wake group. Groups differed neither in the number of times nor the number of minutes rehearsed, Pillai’s trace = .01, *F*(2, 58) = 0.22, *p* = .803, η_p_^2^ = .01.

### Ongoing-task performance

Table 2 shows percentage correct and reaction times on ongoing-task trials. For the analyses of reaction times, we only included correct ongoing-task trials. We excluded trials with reaction times faster than 300 ms and slower than two standard deviations from the individual mean (cf. Rummel & Meiser, 2013). We excluded 4.55% of correct ongoing-task trials due to this correction. A mixed-factorial MANOVA showed that there was no main effect of group on ongoing-task accuracy and reaction times, Pillai’s trace = 0.03, *F*(2, 59) = 0.83, *p* = .440, η_p_^2^ = .03, and no interaction between session number and group, Pillai’s trace = 0.03, *F*(2, 59) = 0.89, *p* = .417, η_p_^2^ = .03. However, it revealed a significant main effect of session number, Pillai’s trace = 0.43, *F*(2, 59) = 22.26, *p* < .001, η_p_^2^ = .43. Follow-up ANOVAS revealed practice effects with higher accuracy in the second than the first session, *F*(1, 60) = 6.33, *p* = .015, η_p_^2^ = .10, and faster reaction times in the second than the first session, *F*(1, 60) = 26.78, *p* < .001, η_p_^2^ = .31.

### PM performance

**Fig. 3:**
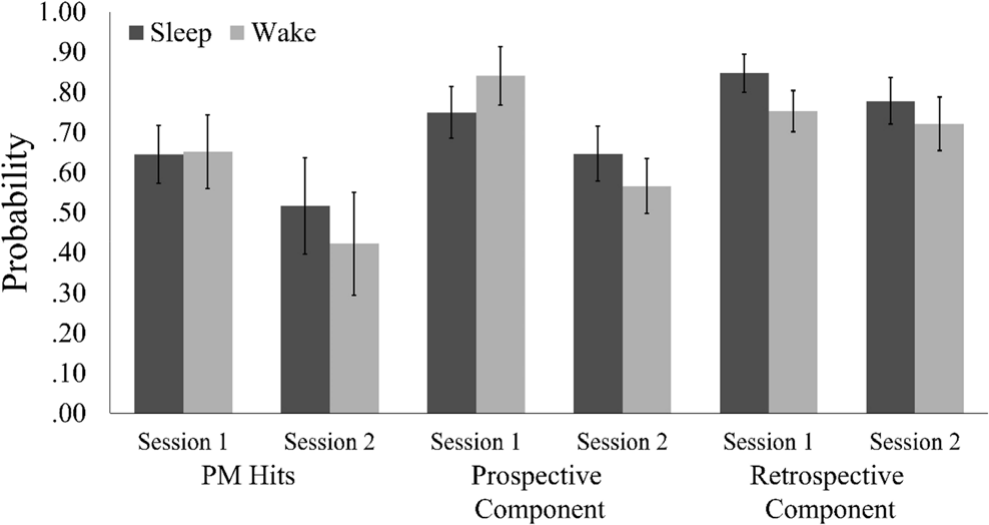
Mean prospective-memory (PM) hit rate and model estimates for the prospective and the retrospective components of PM for the sleep and the wake group in the two sessions. Error bars represent 95% confidence intervals

We measured PM performance as PM hit rate, which is the proportion of PM trials correctly responded to. Belated PM responses, which occurred during the presentation of the colored rectangles of the following trial, were considered as correct PM responses. 17.90% of PM hits were belated.^1^ ANOVA showed a main effect of session number on PM hit rate, *F*(1, 60) = 12.52, *p* = .001, η_p_^2^ = .17. Figure 3 shows that PM hit rate declined expectedly from the first session (short retention interval) to the second session (long retention interval). There was no main effect of group, *F*(1, 60) = 0.68, *p* = .414, η_p_^2^ = .01, and no interaction, *F*(1, 60) = 0.98, *p* = .327, η_p_^2^ = .02.

Hypothesis 3b was that if participants in the sleep group benefited from a consolidated intention–context association after sleep, then in the second session, fewer participants in the sleep group than the wake group should never press the PM key during the ongoing task. As the dependent variable was categorical (the participant did or did not press the PM key at least once during the ongoing task), we computed a logistic regression with session number, group, and their interaction as predictors, and occurrence of at least one PM key press as dependent variable. Session number predicted PM key press, χ^2^(1) = 9.33, *p* = .002; the number of participants who never pressed the PM key increased from the first session (*n*_sleep_ = 0, *n*_wake_ = 1) to the second session (*n*_sleep_ = 4, *n*_wake_ = 6). However, neither group nor interaction between group and session number predicted PM key pressing, χ^2^(1) = 0.91, *p* = .341, and χ^2^(1) = 0.91, *p* = .340, respectively. This does not support the influence of a consolidated intention–context association on PM key presses.

### Multinomial modelling

**Fig. 4:**
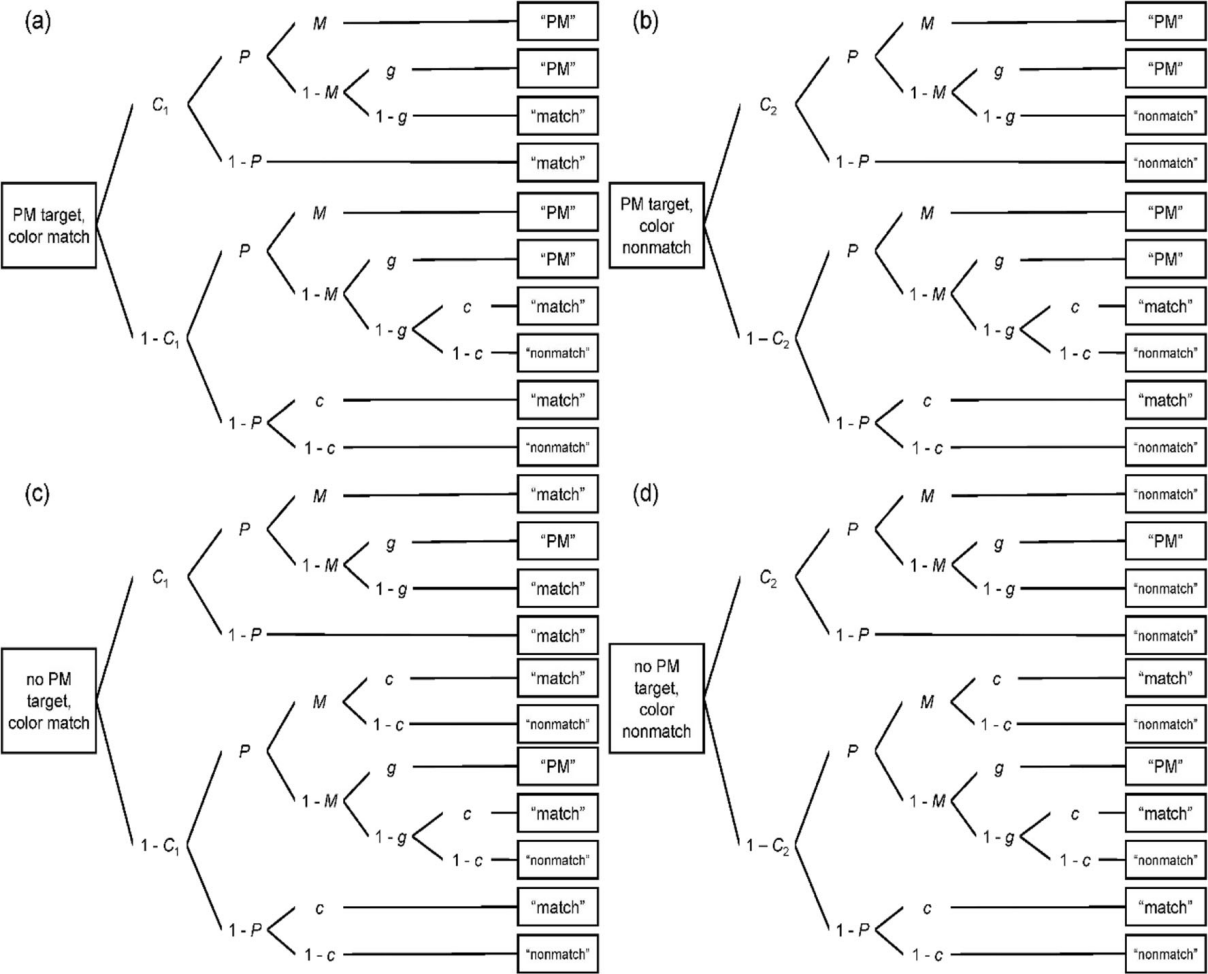
The multinomial model of event-based prospective memory. *Note.* PM = prospective memory, *P* = prospective component, *M* = retrospective component, *g* = probability to guess that the word is a PM target, *C*_1_ = probability to detect a color match, *C*_2_ = probability to detect that colors do not match, *c* = probability to guess that colors match. Adapted from “A Multinomial Model of Event-Based Prospective Memory” by Smith & Bayen, 2004, *Journal of Experimental Psychology: Learning, Memory, and Cognition, 30*(4), p. 758 (10.1037/0278-7393.30.4.756). Copyright 2004 by the American Psychological Association

PM hit rate confounds the prospective and retrospective components of PM; thus, we used the MPT model of event-based PM (Smith & Bayen, 2004) to disentangle these components. Figure 4 illustrates the model.

As shown on the right-hand side of each tree of Fig. 4, participants have three response options on each trial: They may indicate that the colors match (“match”), that the colors do not match (“nonmatch”), or that the word was a PM target (“PM”). As indicated on the left-hand side of the trees, there are four possible item types during the task: (a) PM target, match; (b) PM target, nonmatch; (c) no PM target, match; and (d) no PM target, nonmatch. Three response options for each of four trial types result in a total of 12 response categories.

Tree (a) represents color-match trials with PM targets. With probability *C*_1_, participants detect the color match. They may further remember that they had an intention with probability *P* (prospective component). If they additionally recognize the PM target, with probability *M* (retrospective component), they answer “PM.” If they do not recognize the PM target (with probability 1 − *M*), they guess whether the word is a target (with probability *g*) or not (1 − *g*) and answer “PM” or “match,” respectively. If participants detect the color match (with probability *C*_1_), but do not remember having an intention (1 − *P*), they answer “match.” However, participants may not detect the color match (with probability 1 − *C*_1_). They have an intact prospective component with probability *P* and may recognize the PM target (with probability *M*), resulting in a “PM” response. If participants do not recognize the PM target (1 − *M*), they guess if the word was a target (*g*) or not (1 − *g*). If participants guess that the word was a target, they answer “PM”. However, if participants guess that the word was not a target, they must guess whether the colors match (with probability *c*) or not (1 − *c*), answering “match” or “nonmatch” accordingly, because they fail to detect the color match. If participants do not detect the color match (1 − *C*_1_) and do not remember having an intention (1 − *P*), they also guess whether the colors match (*c*) or not (1 − *c*).

The other trees represent different trial types, but follow the same logic. Trees (b) and (d) represent nonmatch trials with PM targets or distractors, respectively, and include parameter *C*_2_, the probability of detecting a color nonmatch. Trees (c) and (d) represent trials with distractors. In these trees, *M* is the probability that the participant recognizes the distractor. In such a case, the participant will not give the “PM” response, but will respond “match” or “nonmatch” depending on whether the participant detects or guesses a match or a nonmatch.

To achieve model identifiability, we restricted the guessing parameters assuming that participants matched the actual probabilities in the task (*probability matching*; Smith & Bayen, 2004). We thus set parameter *c* equal to .50 (proportion of color matches), and *g* equal to .09 (proportion of PM targets).

The model was validated for nonfocal PM tasks (Horn et al., 2011; Rummel et al., 2011; Smith & Bayen, 2004) and has been applied frequently (Arnold & Bayen, 2019; Arnold, Bayen, & Böhm, 2015; Arnold, Bayen, & Smith, 2015; Böhm et al., 2020a; Pavawalla et al., 2012; Schnitzspahn et al., 2012; Smith et al., 2010; Smith et al., 2014; Smith & Bayen, 2005, 2006; Walter & Bayen, 2016; Wesslein et al., 2014; Zhang et al., 2017).

We expected interactions of group and session number on model parameters. Interactions can be tested by reparametrizing the MPT model (Knapp & Batchelder, 2004). The reparametrized model includes reduction rates for parameters *P* (β_P_) and *M* (β_M_)*.* The reduction rate represents the percentage of the parameter estimate for the first session that remains in the second session. Thus, the smaller the reduction rate, the larger the decline from the first to the second session.

Parameters were estimated via maximum-likelihood estimation (Hu & Batchelder, 1994) based on the aggregated response frequencies that are listed in the supplementary material. We used the log-likelihood ratio statistic *G*^2^, which is asymptotically chi-square distributed, as the goodness-of-fit statistic. The joint MPT model fit the data, *G*^2^(16) = 25.05, *p* = .069. For technical details, see Hu and Batchelder (1994) or Smith and Bayen (2004). Figure 3 shows the parameter estimates. We report analyses involving model-based ongoing-task parameters in the supplementary material.

#### Retrospective component

For the retrospective component (model parameter *M*), we had hypothesized a significant decline from the first to the second session due to different lengths of the retention intervals for the PM targets (Hypothesis 1c). The retrospective component did not significantly decrease from the first to the second session in the sleep group, ∆*G*^2^(1) = 3.32, *p* = .068, and in the wake group, ∆*G*^2^(1) = 0.54, *p* = .463. We had also hypothesized an interaction of group and session number due to presumed effects of sleep on retrospective-memory consolidation (Hypotheses 1d and 1e). However, there was no such interaction, Δ*G*^2^(1) = 0.06, *p* = .805, as reduction rates were comparable in both groups (wake: β_M_ = .96, 95% CI [.85, 1.07]; sleep: β_M_ = .92, 95% CI [.83, 1.00]). In the first session, there were group differences in the retrospective component, Δ*G*^2^(1) = 6.92, *p* = .009, with the sleep group showing higher probability of discriminating target and distractor items. Thus, the retrospective component was better in the evening suggesting a time-of-day effect (contradicting Hypothesis 1a). In the second session, the two groups did not differ, Δ*G*^2^(1) = 1.60, *p* = .206, contradicting Hypothesis 1b.

#### Prospective component

We had hypothesized that the prospective component (model parameter *P*) would decline from the first to the second session (Hypotheses 2c and 4c). There was indeed a significant decline in the prospective component in both the sleep group, ∆*G*^2^(1) = 4.55, *p* = .033, and the wake group, ∆*G*^2^(1) = 28.26, *p* < .001.

Recall that for the prospective component of PM, different mechanisms of sleep effects lead to different hypotheses regarding types of interactions: If intention*–*context associations were consolidated during sleep, an ordinal interaction of group and session number was expected (Hypothesis 2e). If in addition, attention was refreshed by sleep, a hybrid interaction was expected (Hypothesis 4e). There was indeed an interaction of group and session number in the prospective component, Δ*G*^2^(1) = 7.38, *p* = .007, with a stronger decrease in the wake group (β_P_ = .67, 95% CI [.57, .77]) than in the sleep group (β_P_ = .86, 95% CI [.74, .98]) indicating a hybrid interaction. The group differences in the first versus second session were in different directions (see Fig. 3), as predicted by the refreshed-attention hypotheses. That is, in the first session, the prospective component was descriptively higher in the wake group (Hypothesis 4a), whereas in the second session, the prospective component was descriptively higher in the sleep group (Hypothesis 4b). However, in neither session did the predicted group differences reach significance, first session: Δ*G*^2^(1) = 3.47, *p* = .062; second session: Δ*G*^2^(1) = 2.60, *p* = .107.

Effects of sleep on refreshing of attention alone would lead to equal group differences in the prospective component in both sessions, albeit in different directions (Hypotheses 4a and 4b). If, however, a combination of both intention*–*context consolidation during sleep and refreshing of attention during sleep accounted for the interaction, we would expect the difference between the sleep and the wake group in the first session to be smaller than the difference in the second session. We therefore performed an additional analysis to test for differences in group differences. For this test, we again reparametrized the MPT model according to Knapp and Batchelder (2004) so that it included two reduction rates that indicate the difference in the prospective component between the sleep and the wake group in each session (first session: β_PS1_; second session: β_PS2_). In the first session, the reduction rate can be interpreted as the percentage of the prospective-component estimate that the sleep group had in comparison to the wake group. In the second session, the reduction rate can be interpreted as the percentage of the prospective-component estimate that the wake group had in comparison to the sleep group. The larger the difference in the prospective component between both groups in each session, the smaller the reduction rate in this session should be. Thus, if the difference between both groups is larger for one session as compared with the other, this should lead to a statistically significant difference when comparing both reduction rates. However, the reduction rates β_PS1_ (β_PS1_ = .89, 95% CI [.78, > .99]) and β_PS2_ (β_PS2_ = .88, 95% CI [.73, 1.02]) did not differ, ∆*G*^2^(1) = 0.03, *p* = .861. The pattern of results thus constitutes a hybrid interaction that excludes a contribution of sleep effects on intention*–*context consolidation to the prospective component.

## Discussion

To determine which component of PM benefits from sleep and to discern the mechanisms that drive effects, we had participants perform PM tasks before and after one night’s sleep or a day of wakefulness. We disentangled the retrospective and prospective components of PM via MPT modeling. We will discuss results for the retrospective component first, then for the prospective component.

In the first session, the retrospective component of PM was better in the evening than in the morning, suggesting a time-of-day effect (contradicting Hypothesis 1a). Although the mean score on the MEQ (see Table 2) indicated intermediate chronotype and not evening chronotype, a time-of-day effect with a better retrospective component in the evening is still conceivable. In a variety of cognitive domains, young adults’ performance improved over the day as the synchrony between task timing and their preferred time-of-day increased (Hasher et al., 2002; Hasher et al., 1999; May, 1999; May & Hasher, 1998; May et al., 2005; May et al., 1993; for an overview, see Schmidt et al., 2007). However, only few studies were targeted at time-of-day effects on recognition memory, with mixed findings. In line with the present results, Maylor and Badham (2018) showed that young adults performed better on recognition tasks in the evening than in the morning, corresponding with their preferred time of day. Also, May et al. (1993) found better recognition memory in the afternoon than the morning in young adults who were mostly neutral chronotypes. In contrast, other studies did not find a time-of-day effect on recognition (Fenn et al., 2009; Intons-Peterson et al., 1999; Murphy et al., 2007). Thus, the time-of-day effect we found in the retrospective recognition component of the PM task supports the notion that recognition memory is dependent on daily fluctuations with better recognition in the evening for young adults, as suggested by some previous studies.

In the second session, we expected the retrospective component of PM to be better in the sleep group compared with the wake group, based on the literature on sleep-related consolidation of retrospective memory (Hypothesis 1b). We found no difference, however. The wake group had their second test in the evening, thereby possibly benefitting from the time-of-day effect. Thus, the time-of-day effect in the wake group may have masked a sleep effect on the retrospective component. This suggests that the size of time-of-day effects is comparable to the size of sleep effects on recognition. In fact, a study by Koulack (1997) indicated that time-of-day effects may be comparable in size to sleep effects in recognition. However, thorough meta-analyses would be needed to determine the relative size of time-of-day and sleep effects on recognition.

We were most interested in the prospective component, as it is the unique characteristic of PM. As expected, the prospective component decreased as the length of the retention interval increased (Hypotheses 2c and 4c). This decrease was more pronounced in the wake group than in the sleep group (Hypotheses 2d and 4d). While the prospective component did not differ significantly between the groups at either session, the direction of the effect of group changed, and the effect sizes did not differ significantly between sessions. Thus, the pattern of results reflects a hybrid interaction, suggesting a benefit from improved attention in the morning (contrary to Hypothesis 2e, but in line with Hypothesis 4e). In the first session, the wake group had a descriptive advantage over the sleep group. In the second session, this advantage reversed and the sleep group showed a descriptive advantage over the wake group. We statistically determined that the size of the group differences in the first and second session did not differ; it thus seems that consolidation of the intention*–*context association via sleep did not play a role. This conclusion is also supported by the numbers of participants who never pressed the PM key during the second session: There was no interaction between session number and group, indicating that sleep did not influence the number of people who never pressed the PM key in the second session (contrary to Hypothesis 3d). However, such interaction would have been expected if the intention–context association had been consolidated during sleep.

Sleep selectively consolidates weakly encoded information (Drosopoulos et al., 2007; Ekstrand, 1967; Kuriyama et al., 2004; Schapiro et al., 2018), which may explain why we did not identify the consolidation of intention–context associations as a driving mechanism behind the sleep effect on PM. Perhaps performing the PM task during the first session led to a strong encoding of the intention–context association in both groups, and this association was consequently not consolidated to greater degree during a night of sleep than during a day of wakefulness.

We found neither effects of sleep nor of time of day on PM hit rate. This stands in contrast to prior evidence of sleep improving overall PM performance (cf. Leong, Cheng, et al., 2019) and time-of-day effects on PM (e.g., Barner et al., 2019). During the first session, the prospective and the retrospective components had opposite effects (albeit this effect was not significant in the prospective component) so that their effects on PM hits traded off, explaining the null finding in PM hit rate. It is an important advantage of MPT modeling that model parameters may reveal effects that are not detectable in behavioral data due to trade-offs (see also Bayen et al., 2006; Groß & Bayen, 2017; Pavawalla et al., 2012). This is possible because MPT model parameters measure latent cognitive processes that jointly contribute to human behavior. This highlights the importance of distinguishing between the prospective and the retrospective components in studies of effects of sleep on PM.

In the present study, we used a within-subjects design to control for time-of-day effects. One may object that such a design could lead to interference effects, which have also been shown to be affected by sleep (e.g., Abel & Bäuml, 2014), because participants had to retrieve two different sets of PM targets in the two sessions. However, we deem it unlikely that interference played a role in our PM task. In one of our previous studies (Böhm et al., 2020a), participants performed a PM task embedded in an ongoing color-matching task in three consecutive blocks, each with new PM target words. In unpublished analyses of these data (Böhm et al., 2020b), we found that even three consecutive blocks of PM tasks did not induce interference effects. On the contrary, PM performance was even slightly better on the later PM blocks. If there were interference effects in PM tasks, these should have increased the difference between the sleep and the wake group in the second session of our study as sleep has been found to promote resistance to proactive and retroactive interference (Abel & Bäuml, 2014; Alger et al., 2012; Drosopoulos et al., 2007; Ellenbogen et al., 2009; Ellenbogen et al., 2006; Sheth et al., 2012; but see Pöhlchen et al., 2020). However, the group differences were equal in the first and second session. Overall, we deem it unlikely that our findings are confounded by interference effects.

Future studies of PM and sleep may use objective sleep measures, such as polysomnography in a sleep laboratory, to measure sleep that takes place during a retention interval. Subjective sleep diaries have been shown to differ from objective measures (e.g., Kaplan et al., 2017). Thus, objective sleep measures would allow researchers to more precisely assess sleep during the retention interval and to analyze relationships between different sleep stages and PM. As our aim was simply to assess whether participants had slept during the retention interval and to ensure a priori group equivalence, a subjective sleep measure was sufficient for our purpose.

Overall, our study showed that sleep benefits the prospective component, which is the unique characteristic of PM. Thus, we can conclude that the effect of sleep on PM in previous studies likely did not only arise from the established effect of sleep on retrospective memory. The benefit to the prospective component was attributable to refreshed attentional resources, but not to consolidated intention–context associations.

The present findings could help to prevent PM failures in everyday life. As the prospective component may benefit from sleep in the morning, setting reminders for intentions to be performed in the evening may be helpful in daily life. For instance, women should set an alarm to take oral contraceptives in the evening in order to prevent failures of the prospective component of PM.

## Supplementary Information

ESM 1 (DOCX 26 kb)

## Footnotes

1 There was no main effect of group or session number on the percentage of belated PM responses, and these factors did not interact.
