# Supplementary material for: Nighttime sleep benefits the prospective component of prospective memory
Source: Mem Cognit. 2021 Jun 11;49(8):1690–704. doi: 10.3758/s13421-021-01187-w (PMC8563623; doi:10.3758/s13421-021-01187-w)
Supplement: Supplementary file 1 — (DOCX 26 kb) [file 13421_2021_1187_MOESM1_ESM.docx]

**Nighttime Sleep Benefits the Prospective Component of Prospective Memory**

**Online Supplement**

Mateja F. Böhm, Ute J. Bayen, and Reinhard Pietrowsky

Heinrich-Heine-Universität Düsseldorf

**Aggregated Response Frequencies^[[1]](#footnote-1)^**

**Table 1**

*Response Frequencies as a Function of Group and Session, Aggregated across Participants*

|  |  | Sleep Group | | | | Wake Group | | | |
| --- | --- | --- | --- | --- | --- | --- | --- | --- | --- |
| Item type | Response | Session 1 | | Session 2 | | Session 1 | | Session 2 | |
| Target, color match | “match” | 47 |  | 59 |  | 36 |  | 76 |  |
|  | “no match” | 10 |  | 11 |  | 18 |  | 14 |  |
|  | “PM” | 98 |  | 85 |  | 101 |  | 65 |  |
| Target,  no color match | “match” | 8 |  | 4 |  | 5 |  | 6 |  |
|  | “no match” | 45 |  | 76 |  | 49 |  | 83 |  |
|  | “PM” | 102 |  | 75 |  | 101 |  | 66 |  |
| No target,  color match | “match” | 1,211 |  | 1,270 |  | 1,087 |  | 1,194 |  |
|  | “no match” | 329 |  | 261 |  | 422 |  | 337 |  |
|  | “PM” | 10 |  | 19 |  | 41 |  | 19 |  |
| No target,  no color match | “match” | 129 |  | 108 |  | 91 |  | 117 |  |
|  | “no match” | 1,399 |  | 1,421 |  | 1,442 |  | 1,408 |  |
|  | “PM” | 22 |  | 21 |  | 17 |  | 25 |  |
| *Note.* PM = prospective memory | | | | | | | | | |

**Multinomial-Model Based Analyses to Compare Ongoing-Task Parameters**

In the sleep group, parameter *C*_1_ increased from the first session (*C*_1_ = .58, 95% CI [.54, .62]) to the second session (*C*_1_ = .66, 95% CI [.62, .70]), ∆*G*^2^(1) = 9.31, *p* = .002. Similarly, in the wake group, parameter *C*_1_ increased from the first session (*C*_1_ = .44, 95% CI [.39 - .48]) to the second session (*C*_1_ = .57, 95% CI [.53, .61]), ∆*G*^2^(1) = 18.01, *p* < .001. Parameter *C*_1_ was higher in the sleep group than in the wake group in the first session, ∆*G*^2^(1) = 20.42, *p* < .001, and in the second session, ∆*G*^2^(1) = 11.26, *p* < .001.

In the sleep group, parameter *C*_2_ did not change significantly from the first session (*C*_2_ = .83, 95% CI [.80, .85]) to the second session (*C*_2_ = .86, 95% CI [.84, .89]), ∆*G*^2^(1) = 3.22, *p* = .073. Similarly, in the wake group, parameter *C*_2_ did not change significantly from the first session (*C*_2_ = .88, 95% CI [.86, .90]) to the second session (*C*_2_ = .85, 95% CI [.82, .87]), ∆*G*^2^(1) = 3.11, *p* = .078. Parameter *C*_2_ was higher in the wake group than in the sleep group in the first session, ∆*G*^2^(1) = 7.99, *p* = .005. However, the two groups did not differ regarding parameter *C*_2_ in the second session, ∆*G*^2^(1) = 0.52, *p* = .471.

1. Response frequencies for individual participants are available at the Open Science Framework: https://osf.io/83bdn/?view_only=50e3e142561e49ae95839dedd00f188a [↑](#footnote-ref-1)
